# Supplementary material for: Monitoring Pharmaceuticals and Personal Care Products in Drinking Water Samples by the LC-MS/MS Method to Estimate Their Potential Health Risk
Source: Molecules. 2023 Aug 5;28(15):5899. doi: 10.3390/molecules28155899 (PMC10421426; doi:10.3390/molecules28155899)
Supplement: Supplementary file 1 [file molecules-28-05899-s001.zip › molecules-2507844-supplementary.pdf]

molecules MDPI

Table S1. List of analytical standards of drugs included in the validation and their characteristics - CAS number, summary formula, relative molecular weight and structure.

	Analyt	CAS	Summary formula (Relative molecular weight)	Structure	Supplier
1	**Anastrozole**	120511-73-1	$C_{17}H_{19}N_5$ (293.4)	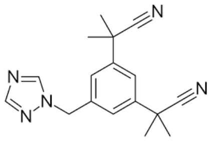	Merck
2	**Atenolol**	29122-68-7	$C_{14}H_{22}N_2O_3$ (266.3)	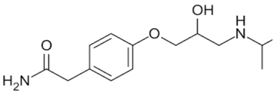	Merck
3	**Azathioprine**	446-86-6	$C_9H_7N_7O_2S$ (277.3)	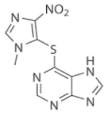	Sigma - Aldrich
4	**Bezafibrate**	41859-67-0	$C_{19}H_{20}ClNO_4$ (361.8)	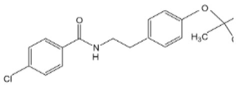	Neochema
5	**Buprenorphine**	52485-79-7	$C_{29}H_{41}NO_4$ (467.6)	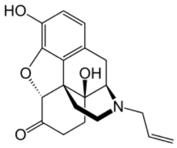	Chromservis
6	**Butorphanol tartrate**	200-659-6	$C_{21}H_{29}NO_2$ (327.5)	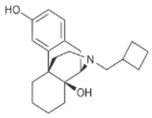	Merck
7	**Caffeine**	58-08-2	$C_8H_{10}N_4O_2$ (194.2)	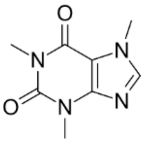	Neochema
8	**Capecitabine**	154361-50-9	$C_{15}H_{22}FN_3O_6$ (359.4)	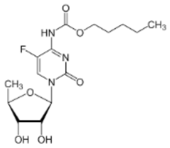	Merck
9	**Carbamazepine**	298-46-4	$C_{15}H_{12}N_2O$ (236.3)	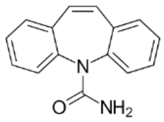	Neochema
10	**Citalopram hydrobromide**	59729-33-8	$C_{20}H_{21}FN_2O \cdot HBr$ (323.1)	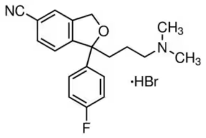	Neochema

	Analyt	CAS	Summary formula (Relative molecular weight)	Structure	Supplier
11	**Clofibric acid**	882-09-7	$C_{10}H_{11}ClO_3$ (214.7)	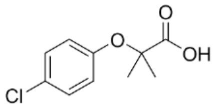	Neochema
12	**Cyclobenzaprine hydrochloride**	6202-23-9	$C_{20}H_{21}N\cdot HCl$ (311.9)	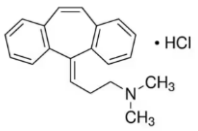	Chromservis
13	**Cyclophosphamide monohydrate**	50-18-0	$C_7H_{15}Cl_2N_2O_2P \cdot H_2O$ (279.1)	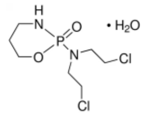	Neochema
14	**Diazepam**	439-14-5	$C_{16}H_{13}ClN_2O$ (284.7)	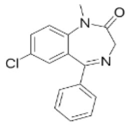	Chromservis
15	**Diclofenac sodium**	15307-79-6	$C_{14}H_{11}Cl_2NO_2$ (296.2)	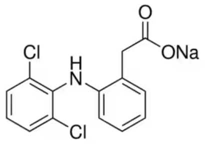	Neochema
16	**Enalapril maleate**	76095-16-4	$C_{20}H_{28}N_2O_5\cdot C_4H_4O_4$ (492.5)	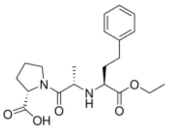	Neochema
17	**Fluoxetine hydrochloride**	59333-67-4	$C_{17}H_{18}F_3NO$ (345.8)	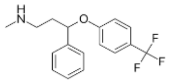	Neochema
18	**Flutamide**	13311-84-7	$C_{11}H_{11}F_3N_2O_3$ (276.2)	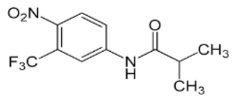	Merck
19	**Furosemide**	54-31-9	$C_{12}H_{11}ClN_2O_5S$ (308.7)	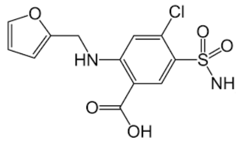	Neochema
20	**Gabapentin**	60142-96-3	$C_9H_{17}NO_2$ (171.2)	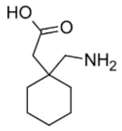	Neochema

	Analyt	CAS	Summary formula (Relative molecular weight)	Structure	Supplier
21	**Gemfibrozil**	25812-30-0	$C_{15}H_{22}O_3$ (250.3)	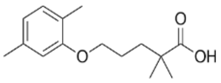	Neochema
22	**Hydrochlorothiazide**	58-93-5	$C_7H_8ClN_3O_4S_2$ (297.7)	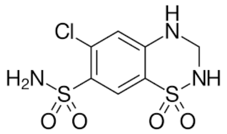	Neochema
23	**Chloramphenicol**	56-75-7	$C_{11}H_{12}Cl_2N_2O_5$ (297.7)	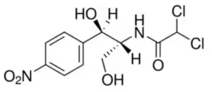	Neochema
24	**Ifosfamide**	3778-73-2	$C_7H_{15}Cl_2N_2O_2P$ (261.1)	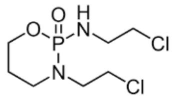	Neochema
25	**Indomethacin**	53-86-1	$C_{19}H_{16}ClNO_4$ (357.8)	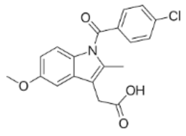	Neochema
26	**Iomeprol**	78649-41-9	$C_{17}H_{22}I_3N_3O_8$ (777.1)	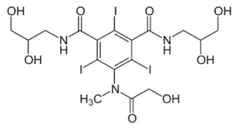	Neochema
27	**Iopamidol**	60166-93-0	$C_{17}H_{22}I_3N_3O_8$ (777.1)	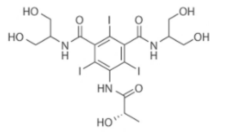	Neochema
28	**Iopromide**	73334-07-3	$C_{18}H_{24}I_3N_3O_8$ (791.1)	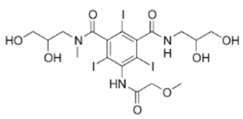	Neochema
29	**Ketoprofen**	22071-15-4	$C_{16}H_{14}O_3$ (254.3)	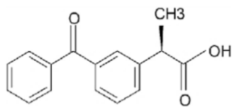	Neochema
30	**Lincomycin hydrochloride**	154-21-2	$C_{18}H_{34}N_2O_6S$ (406.5)	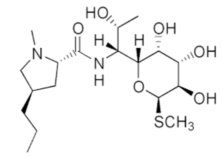	Neochema

	Analyt	CAS	Summary formula (Relative molecular weight)	Structure	Supplier
31	**Loperamide hydrochloride**	34552-83-5	$C_{29}H_{33}ClN_2O_2 \cdot HCl$ (477.0)	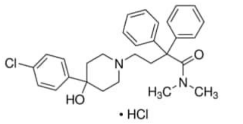	Chromservis
32	**Metoprolol tartrate**	37350-58-6	$C_{15}H_{25}NO_3 \cdot 1/2C_4H_6O_6$ (267.4)	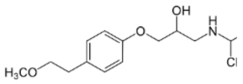	Neochema
33	**Metronidazole**	443-48-1	$C_6H_9N_3O_3$ (171.1)	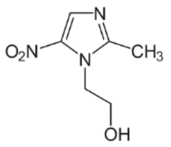	Neochema
34	**Mycophenolate Mofetil**	128794-94-5	$C_{23}H_{31}NO_7$ (433.5)	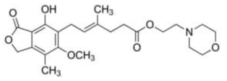	Merck
35	**Naproxen**	22204-53-1	$C_{14}H_{14}O_3$ (230.3)	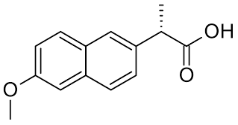	Neochema
36	**Oxazepam**	604-75-1	$C_{15}H_{11}ClN_2O_2$ (286.7)	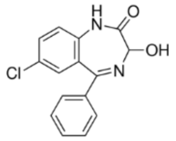	Chromservis
37	**Paclitaxel**	33069-62-4	$C_{47}H_{51}NO_{14}$ (853.9)	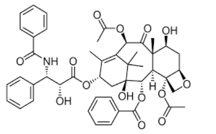	Chromservis
38	**Paracetamol (Acetaminophen)**	103-90-2	$C_8H_9NO_2$ (151.2)	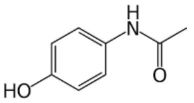	Neochema
39	**Piroxicam**	36322-90-4	$C_{15}H_{13}N_3O_4S$ (331.4)	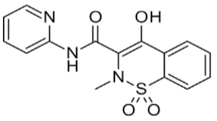	Neochema
40	**Propranolol hydrochloride**	525-66-6	$C_{16}H_{22}ClNO_2 \cdot HCl$ (259.3)	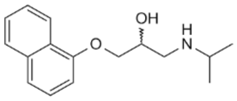	Neochema

	Analyt	CAS	Summary formula (Relative molecular weight)	Structure	Supplier
41	**Salbutamol**	18559-94-9	$C_{13}H_{21}NO_3$ (239.3)	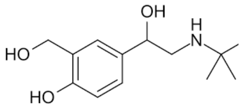	Neochema
42	**Sertraline hydrochloride**	79617-96-2	$C_{17}H_{17}Cl_2N \cdot HCl$ (306.2)	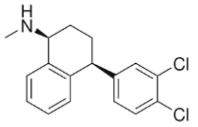	Merck
43	**Sotalol hydrochloride**	959-24-0	$C_{12}H_{20}N_2O_3S \cdot HCl$ (308.8)	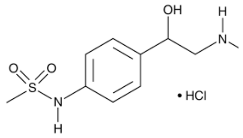	Neochema
44	**Sulfamethazine**	57-68-1	$C_{12}H_{14}N_4O_2S$ (278.3)	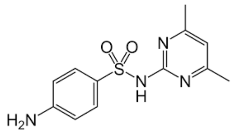	Neochema
45	**Sulfamethoxazole**	723-46-6	$C_{10}H_{11}N_3O_3S$ (253.3)	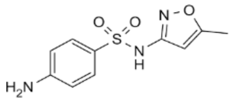	Neochema
46	**Terbutaline**	23031-25-6	$C_{12}H_{19}NO_3$ (225.3)	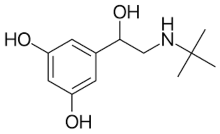	Neochema
47	**Thebaine**	115-37-7	$C_{19}H_{21}NO_3$ (311.4)	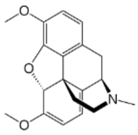	TEVA
48	**Tramadol hydrochloride**	36282-47-0	$C_{16}H_{25}NO_2 \cdot HCl$ (263.4)	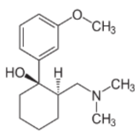	Neochema
49	**Trimethoprim**	738-70-5	$C_{14}H_{18}N_4O_3$ (290.3)	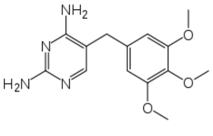	Neochema
50	**Valsartan**	137862-53-4	$C_{24}H_{29}N_5O_3$ (435.5)	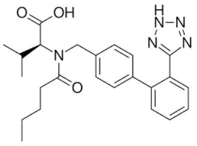	Neochema

	Analyt	CAS	Summary formula (Relative molecular weight)	Structure	Supplier
51	**Warfarin**	81-81-2	$C_{19}H_{16}O_4$ (308.3)	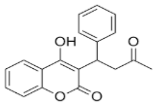	Neochema
52	**Zolpidem hemitartrate**	99294-93-6	$C_{19}H_{21}N_3O$ (307.4)	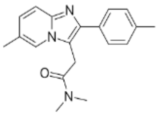	Chromservis

Table S2. List of ISTDs included in the validation and their characteristics - ISTD CAS number, summary formula, relative molecular weight and structure.

	Name	CAS	Summary formula (Relative molecular weight)	Structure	Supplier
1	**Atrazine D5**	163165-75-1	$C_8H_{14}ClN_5$ (220.7)	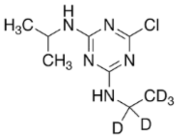	Chromservis
2	**Atrazine-desisopropyl D5**	1189961-78-1	$C_5H_8ClN_5$ (178.63)	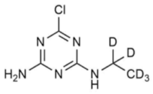	Chromservis
3	**Caffeine-13C**	78072-66-9	$^{13}C_3C_5H_{10}N_4O_2$ (197.17)	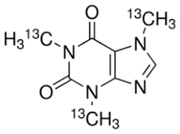	Neochema
4	**Carbamazepine D10**	132183-78-9	$C_{15}D_{10}H_2N_2O$ (246.33)	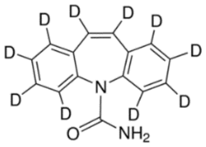	Neochema

	Name	CAS	Summary formula (Relative molecular weight)	Structure	Supplier
5	**Carbendazim D4**	291765-95-2	$C_9H_9N_3O_2$ (195.21)	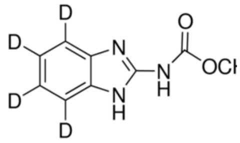	HPST
6	**Citalopram D6 hydrobromide**	1190003-26-9	$C_{20}H_{15}D_6FN_2O$ ·HBr (330.43)	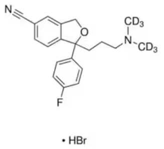	Chromservis
7	**Diclofenac D4**	153466-65-0	$C_{14}H_7D_4Cl_2NO_2$ (300.17)	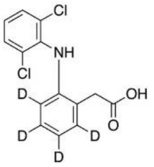	Neochema
8	**Metolachlor D6**	1219803-97-0	$C_{15}H_{16}D_6ClNO_2$ (289.83)	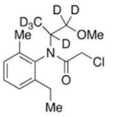	Chromservis
9	**Naproxen 13C D3**	1216704-11-8	$C_{13}{}^{13}CH_{11}D_3O_3$ (234.27)	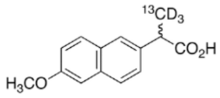	Chromservis
10	**Noreethindrone D6**	-	$C_{20}H_{20}D_6O_2$ (304.46)	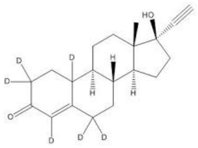	Chromservis
11	**Phosalone D10**	-	$C_{12}H_5ClD_{10}NO_4PS_2$ (377.87)	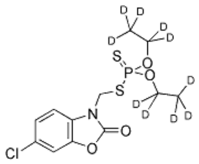	Chromservis
12	**Sulfamethoxazole D4**	1020719-86-1	$C_{10}H_7D_4N_3O_3S$ (257.30)	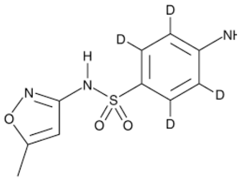	Neochema

	Name	CAS	Summary formula (Relative molecular weight)	Structure	Supplier
13	**Tebuconazole D6**	-	$C_{16}H_{16}ClN_3OD_6$ (313.86)	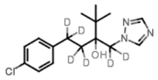	Chromservis

Table S3. Parameters of the MS/MS method (MRM transitions of analytes. collision energy and retention time of analytes) - transitions marked in bold are used as quantification (* designation of precursor ions containing 2 isotopes of chlorine atoms).

Analyte	Ionization	Precursor ions (*m/z*)	Product ions (*m/z*)	Cone voltage (V)	Collision energy (V)	Retention time (t_R)
**Anastrozole**	ESI+	294.1	**225.0**/114.8	30	20/52	4.24
**Atenolol**	ESI+	267.0	**145.0**/190.0	30	23/16	2.55
**Azathioprine**	ESI+	278.0	**141.8**/232.0	30	11/13	2.92
**Bezafibrate**	ESI+	262.0	**139.0**/121.0	30	25/29	4.70
**Buprenorphine**	ESI+	468.3	**396.1**/414.2	30	39/32	3.83
**Butorphanol**	ESI+	328.0	**124.0**/282.0	30	26/29	3.47
**Caffeine**	ESI+	195.0	**138.0**/110.0	30	17/23	2.89
**Capecitabine**	ESI+	360.0	**173.8**/244.0	30	19/10	3.94
**Carbamazepine**	ESI+	237.0	**194.0**/179.0	35	20/35	4.16
**Citalopram**	ESI+	325.2	**109.0**/262.2	30	24/19	3.72
**Clofibric acid**	ESI-	213.0	**127.0**/85.0	30	15/11	4.59
**Cyclobenzaprine**	ESI+	276.0	**216.0**/231.0	30	24/16	3.93
**Cyclophosphamide**	ESI+	261.0	**140.0**/106.0	30	22/18	3.70
**Diazepam**	ESI+	285.0	**154.0**/193.0	30	26/30	4.94
**Diclofenac**	ESI+	294.0/296.0*	**250.0**/252.0	30	11/11	5.21

Analyte	Ionization	Precursor ions (*m*/*z*)	Product ions (*m*/*z*)	Cone voltage (V)	Collision energy (V)	Retention time (t_R)
**Enalapril**	ESI-	377.1	**234.0**/91.0	30	20/55	3.66
**Fluoxetine**	ESI+	310.1	**148.0**/44.0	30	8/7	4.05
**Flutamide**	ESI-	275.0	**202.0**/205.0	30	23/22	5.09
**Furosemide**	ESI-	329.0	**205.0**/285.0	30	22/14	4.06
**Gabapentin**	ESI+	172.2	**154.3**/137.3	25	15/18	2.72
**Gemfibrozil**	ESI-	249.0	**121.0**/127.0	30	15/10	5.47
**Hydrochlorothiazide**	ESI-	296.0	**269.0**/205.0	30	19/22	2.88
**Chloramphenicol**	ESI-	321.0	**152.1**/257.1	30	18/12	3.73
**Ifosfamide**	ESI+	261.0	**92.0**/154.0	30	25/21	3.64
**Indomethacin**	ESI+	358.0	**139.0**/174.0	30	22/11	5.20
**Iomeprol**	ESI+	777.8	**405.0**/531.8	30	38/30	2.46
**Iopamidol**	ESI+	777.8	**387.0**/313.8	30	39/52	1.93
**Iopromide**	ESI+	791.8	**300.0**/572.8	30	56/23	2.65
**Ketoprofen**	ESI+	255.1	**209.0**/105.0	30	15/23	4.67
**Lincomycin**	ESI+	407.0	**126.0**/359.0	35	20/18	2.83
**Loperamide**	ESI+	477.2	**210.1**/266.1	30	45/12	4.29
**Metoprolol**	ESI+	268.2	**116.0**/72.0	30	18/22	3.25
**Metronidazole**	ESI+	172.0	**128.0**/82.0	30	15/21	2.71
**Mycophenolate Mofetil**	ESI+	434.0	**114.0**/195.0	30	24/33	3.82
**Naproxen**	ESI+	231.0	**185.0**/170.0	30	14/29	4.63
**Oxazepam**	ESI+	287.0	**241.0**/269.0	30	23/15	4.30
**Paclitaxel**	ESI+	876.4	**308.1**/591.3	30	27/23	5.08
**Paracetamol (Acetaminophen)**	ESI+	152.0	**110.0**/65.0	30	15/30	2.68

Analyte	Ionization	Precursor ions (*m*/*z*)	Product ions (*m*/*z*)	Cone voltage (V)	Collision energy (V)	Retention time (t_R)
**Piroxicam**	ESI+	332.0	**95.0**/121.0	30	18/25	4.33
**Propranolol**	ESI+	260.0	**116.0**/183.0	30	16/16	3.65
**Salbutamol**	ESI+	240.0	**148.0**/166.0	30	20/12	2.52
**Sertraline**	ESI+	306.0	**159.0**/275.0	30	23/11	4.04
**Sotalol**	ESI+	273.0	**133.0**/213.0	30	27/17	2.52
**Sulfamethazine**	ESI+	279.0	**124.0**/186.0	30	20/18	3.26
**Sulfamethoxazole**	ESI+	254.0	**156.0**/92.0	30	16/26	3.58
**Terbutaline**	ESI+	226.1	**152.0**/107.0	30	16/26	2.47
**Thebaine**	ESI+	312.0	**58.0**/266.0	30	30/40	3.29
**Tramadol**	ESI+	264.0	**58.0**/246.0	30	15/10	3.26
**Trimethoprim**	ESI+	291.0	**230.0**/123.0	39	24/27	2.90
**Valsartan**	ESI+	436.0	**207.0**/235.0	30	28/22	4.72
**Warfarin**	ESI+	309.0	**163.0**/251.0	30	14/22	4.91
**Zolpidem**	ESI+	308.0	**235.0**/263.0	30	33/27	3.44
**Atrazine D5**	ESI+	221.0/223.0*	**179.0**/181.0	35	18/18	4.53
**Atrazine-desisopropyl D5**	ESI+	179.0	**101.0**/137.0	35	18/16	3.10
**Caffeine-13C**	ESI+	198.0	**140.0**/112.0	30	19/22	2.89
**Carbamazepine D10**	ESI+	247.0	**204.0**/201.0	30	21/22	4.14
**Carbendazim D4**	ESI+	196.0	**164.0**/136.0	35	20/28	2.90
**Citalopram D6**	ESI+	331.3	**109.1**/262.2	30	26/19	3.78
**Diclofenac D4**	ESI-	298.0/300.0*	**254.0**/256.0	30	12/12	5.20
**Phosalon D10**	ESI+	378.0	**182.0**/111.0	35	16/38	5.77
**Metolachlor D6**	ESI+	290.0	**258.0**/182.0	35	16/25	5.39

Analyte	Ionization	Precursor ions (*m*/*z*)	Product ions (*m*/*z*)	Cone voltage (V)	Collision energy (V)	Retention time (t_R)
**Naproxen 13C D3**	ESI+	235.0	**189.0**/170.0	30	15/25	4.62
**Noreethindrone D6**	ESI+	305.3	**113.0**/237.2	30	30/21	4.71
**Sulfamethoxazole D4**	ESI+	258.0	**160.0**/96.0	30	16/26	3.57
**Tebuconazole D6**	ESI+	314.0	**71.8**/124.8	30	21/38	5.24

Table S4. List of ISTDs and assigned analytes in MQ and drinking matrices.

ISTD – MQ and drinking water	Analyte
Atrazine D5	Anastrozole
	Bezafibrate
	Capecitabine
	Cyclophosphamide
	Diazepam
	Enalapril
	Flutamide
	Indomethacin
	Oxazepam
	Piroxicam
	Sulfamethazine
Atrazine-desisopropyl D5	Lincomycin
	Paracetamol (Acetaminophen)
Caffeine-13C	Caffeine
	Iopamidol
	Iopromide
Carbamazepine D10	Carbamazepine
	Fluoxetine
	Valsartan
Carbendazim D4	Azathioprine
	Cyklobenzaprine
	Gabapentin
	Chloramphenikol

ISTD – MQ and drinking water	Analyte
	Ifosfamide
	Metronidazole
	Trimethoprim
	Zolpidem
Citalopram D6	Atenolol
	Buprenorphine
	Butorphanol
	Citalopram
	Iomeprol
	Loperamide
	Metoprolol
	Salbutamol
	Sotalol
	Terbutaline
	Thebaine
	Tramadol
Diclofenac D4	Diclofenac
Metolachlor D6	Gemfibrozil
Naproxen 13C D3	Clofibric acid
Noreethindrone D6	Naproxen
	Paclitaxel
	Propranolol
	Sertraline
Phosalone D10	Furosemide
	Ketoprofen
Sulfamethoxazole D4	Mycophenolate mofetil
	Sulfamethoxazole
Tebuconazole D6	Hydrochlorothiazide
	Warfarin

Table S5. Overview of linear range, coefficient of determination (R2) values and working range for individual analytes in drinking water.

Analyte	Linear range [ng L-1]	Coefficient of determination (R^2)	Working range for drinking waters [ng L-1]
**Anastrozole**	2.5 – 500	0.9998	5.0 – 500
**Atenolol**	2.5 – 500	0.9997	10.0 – 500
**Azathioprine**	2.5 – 500	0.9997	10.0 – 500
**Bezafibrate**	2.5 – 1000	0.9999	10.0 – 1000
**Buprenorphine**	2.5 – 1000	0.9994	5.0 – 1000
**Butorphanol**	2.5 – 1000	0.9997	25.0 – 500
**Caffeine**	50.0 – 1000	0.9988	250.0 – 1000
**Capecitabine**	5.0 – 1000	0.9992	250.0 – 1000
**Carbamazepine**	2.5 – 1000	1.0000	5.0 – 1000
**Citalopram**	2.5 – 1000	0.9993	25.0 – 1000
**Clofibric acid**	50.0 – 1000	0.9996	250.0 – 1000
**Cyclobenzaprine**	50.0 – 1000	0.9997	100.0 – 1000
**Cyclophosphamide**	2.5 – 1000	0.9999	10.0 – 1000
**Diazepam**	2.5 – 1000	0.9999	2.5 – 1000
**Diclofenac**	5.0 – 1000	0.9996	50.0 – 1000
**Enalapril**	2.5 – 1000	0.9999	10.0 – 1000
**Fluoxetine**	50.0 – 1000	0.9993	250.0 – 1000
**Flutamide**	2.5 – 1000	0.9999	10.0 – 1000
**Furosemide**	5.0 – 1000	0.9995	25.0 – 1000
**Gabapentin**	50.0 – 1000	0.9997	50.0 – 1000
**Gemfibrozil**	5.0 – 1000	0.9997	25.0 – 1000
**Hydrochlorothiazide**	2.5 – 500	0.9997	25.0 – 500
**Chloramfenikol**	2.5 – 1000	0.9992	25.0 – 1000
**Ifosfamide**	2.5 – 1000	0.9999	10.0 – 1000
**Indomethacin**	2.5 – 1000	0.9999	10.0 – 1000
**Iomeprol**	50.0 – 1000	0.9994	500.0 – 1000
**Iopamidol**	2.5 – 1000	0.9991	25.0 – 1000
**Iopromide**	2.5 – 500	0.9991	25.0 – 500
**Ketoprofen**	2.5 – 1000	0.9998	10.0 – 1000
**Lincomycin**	2.5 – 1000	0.9999	10.0 – 1000
**Loperamide**	50.0 – 1000	0.9997	250.0 – 1000
**Metoprolol**	2.5 – 1000	0.9996	10.0 – 1000
**Metronidazole**	2.5 – 500	0.9999	2.5 – 500
**Mycophenolate Mofetil**	2.5 – 1000	0.9998	25.0 – 1000
**Naproxen**	50.0 – 1000	0.9993	75.0 – 1000/
**Oxazepam**	2.5 – 1000	0.9999	5.0 – 1000
**Paclitaxel**	5.0 – 1000	0.9995	250.0 – 1000
**Paracetamol**	2.5 – 500	0.9998	25.0 – 500
**Piroxicam**	2.5 – 1000	1.0000	5.0 – 1000
**Propranolol**	2.5 – 1000	0.9998	10.0 – 1000

Analyte	Linear range [ng L^{-1}]	Coefficient of determination (R^2)	Working range for drinking waters [ng L^{-1}]
**Salbutamol**	2.5 – 500	0.9997	25.0 – 500
**Sertraline**	50.0 – 1000	0.9990	250.0 – 1000
**Sotalol**	2.5 – 500	0.9996	10.0 – 500
**Sulfamethazine**	2.5 – 1000	0.9999	5.0 – 1000
**Sulfamethoxazole**	2.5 – 1000	0.9999	10.0 – 1000
**Terbutaline**	2.5 – 500	0.9997	25.0 – 500
**Thebaine**	2.5 – 1000	0.9998	25.0 – 1000
**Tramadol**	2.5 – 1000	0.9998	25.0 – 1000
**Trimethoprim**	2.5 – 1000	0.9999	25.0 – 1000
**Valsartan**	2.5 – 1000	0.9999	25.0 – 1000
**Warfarin**	2.5 – 1000	0.9999	10.0 – 1000
**Zolpidem**	2.5 – 500	0.9998	10.0 – 500

Table S6. Summary of repeatability results expressed as RSD (%). average of measured concentrations of a given analyte at one concentration level and standard deviation of individual analytes in MQ water.

Analyte	MQ water					
	L1 (0.01 ng mL^{-1})			L2 (0.1 ng mL^{-1})		
	Average [ng L^{-1}]	SD [ng L^{-1}]	RSD [%]	Average [ng L^{-1}]	SD [ng L^{-1}]	RSD [%]
**Anastrozole**	9.57	0.26	2.69	94.09	2.56	2.72
**Atenolol**	11.47	0.88	7.67	113.00	4.05	3.58
**Azathioprine**	9.87	0.48	4.86	94.84	2.02	2.13
**Bezafibrate**	8.88	0.86	9.67	82.09	6.82	8.31
**Buprenorphine**	12.04	0.40	3.34	113.34	6.63	5.85
**Butorphanol**	11.92	1.12	9.42	115.00	7.19	6.25
**Caffeine**	n.d.*	-	-	95.94	15.60	17.15
**Capecitabine**	n.d.*	-	-	88.04	17.02	19.34
**Carbamazepine**	9.54	0.36	3.80	95.47	1.75	1.83
**Citalopram**	13.39	1.37	10.23	112.12	5.22	4.66
**Clofibric acid**	n.d.*	-	-	66.05	12.59	19.07
**Cyclobenzaprine**	n.d.*	-	-	67.31	7.70	11.44
**Cyclophosphamide**	9.26	0.56	6.05	95.57	2.51	2.63
**Diazepam**	9.79	0.19	1.96	93.73	1.90	2.03
**Diclofenac**	n.d.*	-	-	77.64	10.55	13.59
**Enalapril**	9.89	0.46	4.62	98.87	1.53	1.55
**Fluoxetine**	n.d.*	-	-	93.57	17.08	18.26
**Flutamide**	8.69	0.55	6.31	80.21	9.53	11.88
**Furosemide**	8.14	1.47	18.00	68.79	11.83	17.20
**Gabapentin**	n.d.*	-	-	88.59	2.97	3.36
**Gemfibrozil**	10.59	1.05	9.88	90.92	5.25	5.77
**Hydrochlorothiazide**	n.d.*	-	-	97.86	6.27	6.41

Analyte	MQ water					
	L1 (0.01 ng mL-1)			L2 (0.1 ng mL-1)		
	Average [ng L-1]	SD [ng L-1]	RSD [%]	Average [ng L-1]	SD [ng L-1]	RSD [%]
**Chloramfenikol**	11.42	1.39	12.14	92.78	4.56	4.92
**Ifosfamide**	10.02	0.62	6.16	95.11	2.32	2.44
**Indomethacin**	8.44	0.70	8.28	73.56	8.58	11.66
**Iomeprol**	n.d.*	-	-	87.95	25.87	16.38
**Iopamidol**	9.96	0.92	9.26	103.09	0.92	0.89
**Iopromide**	8.13	1.09	13.46	105.72	5.11	4.83
**Ketoprofen**	7.79	0.58	7.48	76.60	7.72	10.07
**Lincomycin**	10.28	0.64	6.24	100.96	1.68	1.67
**Loperamide**	n.d.*	-	-	145.68	19.76	13.56
**Metoprolol**	11.55	0.90	7.77	103.46	2.72	2.63
**Metronidazole**	9.90	0.19	1.88	98.19	0.74	0.75
**Mycophenolate Mofetil**	8.10	1.50	18.52	86.08	4.92	5.72
**Naproxen**	n.d.*	-	-	79.33	6.37	8.03
**Oxazepam**	9.56	0.32	3.30	94.31	2.40	2.54
**Paclitaxel**	n.d.*	-	-	74.46	12.44	19.29
**Paracetamol**	9.25	1.20	12.96	92.01	1.32	1.43
**Piroxicam**	9.93	0.31	3.09	97.59	1.10	1.12
**Propranolol**	9.42	0.63	6.65	82.26	10.54	16.94
**Salbutamol**	11.96	0.93	7.81	113.13	3.82	3.38
**Sertraline**	n.d.*	-	-	73.65	14.35	19.49
**Sotalol**	10.97	0.81	7.37	107.08	3.48	3.25
**Sulfamethazine**	9.68	0.24	2.49	97.16	1.85	1.91
**Sulfamethoxazole**	9.46	0.55	5.78	97.58	1.44	1.48
**Terbutaline**	11.93	1.37	11.46	117.22	6.27	5.35
**Thebaine**	11.92	1.38	11.61	106.99	5.53	5.17
**Tramadol**	11.54	1.09	9.44	105.59	3.67	3.48
**Trimethoprim**	8.04	1.19	14.81	77.05	8.88	11.52
**Valsartan**	9.09	1.27	14.00	82.81	7.58	9.15
**Warfarin**	8.04	0.51	6.36	80.45	3.35	4.16
**Zolpidem**	9.29	0.68	7.32	88.92	4.02	4.52

n.d. - detection limit of the analyte is higher than the lower concentration level of the spike L1 = 0.01 μg L-1*

Table S7. Summary of repeatability results expressed as RSD (%). average of measured concentrations of a given analyte at one concentration level and standard deviation of individual analytes in drinking water.

Analyte	Drinking water					
	L1 (0.01 ng mL-1)			L2 (0.1 ng mL-1)		
	Average [ng L-1]	SD [ng L-1]	RSD [%]	Average [ng L-1]	SD [ng L-1]	RSD [%]
**Anastrozole**	9.47	0.35	3.66	94.30	1.65	1.75

Analyte	Drinking water					
	L1 (0.01 ng mL^{-1})			L2 (0.1 ng mL^{-1})		
	Average [ng L^{-1}]	SD [ng L^{-1}]	RSD [%]	Average [ng L^{-1}]	SD [ng L^{-1}]	RSD [%]
**Atenolol**	10.75	0.48	4.43	101.98	1.67	1.64
**Azathioprine**	9.39	0.38	4.09	91.88	2.35	2.56
**Bezafibrate**	8.27	0.79	9.57	91.29	6.00	6.57
**Buprenorphine**	8.93	1.03	11.53	90.03	5.83	6.48
**Butorphanol**	9.47	0.32	3.36	94.51	0.99	1.05
**Caffeine**	n.d.*	-	-	66.84	5.50	8.24
**Capecitabine**	n.d.*	-	-	220.97*	15.88	7.19
**Carbamazepine**	10.01	0.30	3.01	95.98	0.96	1.00
**Citalopram**	9.71	0.65	6.65	96.71	4.37	4.52
**Clofibric acid**	n.d.*	-	-	72.59	9.94	13.69
**Cyclobenzaprine**	n.d.*	-	-	113.73	2.64	2.32
**Cyclophosphamide**	9.11	0.51	5.61	95.43	1.84	1.92
**Diazepam**	9.48	0.18	1.91	96.55	1.13	1.17
**Diclofenac**	n.d.*	-	-	82.51	6.30	7.63
**Enalapril**	10.87	0.22	2.03	112.76	1.24	1.10
**Fluoxetine**	n.d.*	-	-	117.24	4.19	3.58
**Flutamide**	8.09	0.99	12.18	85.38	6.96	8.15
**Furosemide**	9.42	0.93	9.90	78.48	9.72	12.39
**Gabapentin**	n.d.*	-	-	104.98	4.19	3.99
**Gemfibrozil**	8.86	1.54	17.37	89.48	5.16	5.77
**Hydrochlorothiazide**	n.d.*	-	-	101.70	8.25	8.11
**Chloramfenikol**	10.40	1.59	15.33	93.80	4.58	4.88
**Ifosfamide**	9.80	0.51	5.17	94.23	1.40	1.48
**Indomethacin**	8.01	1.00	12.43	84.73	6.74	7.96
**Iomeprol**	n.d.*	-	-	255.86*	45.18	17.66
**Iopamidol**	7.04	0.45	6.37	97.52	4.41	4.52
**Iopromide**	7.58	1.23	16.24	118.59	6.36	5.37
**Ketoprofen**	8.74	0.34	3.87	89.44	4.37	4.89
**Lincomycin**	9.66	0.27	2.82	98.26	2.10	2.13
**Loperamide**	n.d.*	-	-	134.31*	1.98	1.48
**Metoprolol**	9.56	0.74	7.69	97.64	2.62	2.69
**Metronidazole**	9.28	0.16	1.70	96.58	0.72	0.74
**Mycophenolate Mofetil**	9.19	0.95	10.34	88.63	3.86	4.35
**Naproxen**	n.d.*	-	-	88.65	9.70	10.94
**Oxazepam**	9.18	0.54	5.93	94.50	2.08	2.20
**Paclitaxel**	n.d.*	-	-	73.22	7.83	10.69
**Paracetamol**	9.44	0.70	7.45	99.41	5.00	5.03
**Piroxicam**	9.28	0.38	4.11	96.09	3.29	3.43
**Propranolol**	9.69	0.68	7.01	99.35	3.50	3.52
**Salbutamol**	10.04	0.12	1.21	104.25	2.20	2.11
**Sertraline**	n.d.*	-	-	116.22	1.66	1.43
**Sotalol**	9.64	0.28	2.93	99.98	1.47	1.47

Analyte	Drinking water					
	L1 (0.01 ng mL^{-1})			L2 (0.1 ng mL^{-1})		
	Average [ng L^{-1}]	SD [ng L^{-1}]	RSD [%]	Average [ng L^{-1}]	SD [ng L^{-1}]	RSD [%]
**Sulfamethazine**	9.75	0.42	4.34	94.80	1.19	1.25
**Sulfamethoxazole**	9.48	0.42	4.42	93.47	1.69	1.81
**Terbutaline**	9.32	0.39	4.18	99.91	2.00	2.00
**Thebaine**	9.19	0.73	7.94	87.18	4.11	4.72
**Tramadol**	9.40	0.60	6.40	92.89	3.24	3.49
**Trimethoprim**	8.96	0.51	5.65	92.89	3.78	4.07
**Valsartan**	7.95	1.06	13.36	94.91	6.68	7.04
**Warfarin**	9.25	0.68	7.34	89.21	2.20	2.46
**Zolpidem**	9.03	0.35	3.93	94.05	1.36	1.45

n.d. - detection limit of the analyte is higher than the lower concentration level of the spike L1 = 0.01 µg L^{-1}*

** The given analytes were determined in raw validation samples at levels that were many times higher than the level of fortification. For these analytes. RSD (%) values were calculated from concentrations determined by repeated spike analysis.*

Table S8. Method accuracy results expressed as recovery for MQ and drinking water.

Analyte	Recovery [%]			
	MQ water		Driking water	
	L1	L2	L1	L2
**Anastrozole**	100.1	102.4	100.3	98.9
**Atenolol**	84.0	96.7	107.3	104.9
**Azathioprine**	104.9	103.1	96.9	94.1
**Bezafibrate**	92.9	89.4	87.6	95.8
**Buprenorphine**	88.1	97.0	89.2	92.6
**Butorphanol**	87.2	98.4	94.6	97.2
**Caffeine**	n.d.*	91.7	n.d.*	70.1
**Capecitabine**	n.d.*	95.8	n.d.*	231.8
**Carbamazepine**	97.7	99.5	101.4	97.3
**Citalopram**	98.0	95.9	96.9	99.4
**Clofibric acid**	n.d.*	84.9	n.d.*	75.7
**Cyclobenzaprine**	n.d.*	73.1	n.d.*	116.5
**Cyclophosphamide**	96.9	104.0	96.5	100.1
**Diazepam**	102.4	102.0	99.2	101.3
**Diclofenac**	n.d.*	97.0	n.d.*	86.5
**Enalapril**	103.5	107.6	115.2	118.3
**Fluoxetine**	n.d.*	97.5	n.d.*	118.9
**Flutamide**	91.0	87.3	85.7	89.6
**Furosemide**	90.2	81.6	112.5	94.1
**Gabapentin**	n.d.*	96.3	116.9	107.5
**Gemfibrozil**	108.9	97.3	92.2	93.5

Analyte	Recovery [%]			
	MQ water		Driking water	
	L1	L2	L1	L2
Hydrochlorothiazide	n.d.*	104.9	n.d.*	109.0
Chloramfenikol	121.3	100.8	107.3	96.0
Ifosfamide	106.5	103.4	101.1	96.5
Indomethacin	88.3	80.1	84.8	88.9
Iomeprol	n.d.*	135.1	n.d.*	263.1
Iopamidol	103.4	104.0	74.0	102.3
Iopromide	84.4	106.6	79.7	124.4
Ketoprofen	86.3	90.8	104.4	107.3
Lincomycin	105.2	105.3	101.8	102.1
Loperamide	n.d.*	124.6	n.d.*	138.1
Metoprolol	84.5	88.5	95.4	100.4
Metronidazole	105.2	106.7	95.7	98.9
Mycophenolate Mofetil	83.3	88.7	96.1	92.8
Naproxen	n.d.*	98.4	n.d.*	94.2
Oxazepam	100.0	102.7	97.2	99.1
Paclitaxel	n.d.*	82.9	n.d.*	76.3
Paracetamol	94.6	95.9	99.4	103.3
Piroxicam	103.9	106.2	98.3	100.8
Propranolol	14.7	80.1	88.8	103.6
Salbutamol	87.5	96.8	100.2	107.2
Sertraline	n.d.*	94.7	n.d.*	121.2
Sotalol	80.3	91.6	96.2	102.8
Sulfamethazine	101.3	105.8	103.3	99.4
Sulfamethoxazole	97.2	100.6	99.1	97.9
Terbutaline	87.3	100.3	93.0	102.7
Thebaine	87.2	91.5	91.7	89.6
Tramadol	84.5	90.3	93.8	95.5
Trimethoprim	85.5	83.7	92.4	95.1
Valsartan	93.1	86.3	80.6	96.2
Warfarin	81.7	86.3	96.1	95.6
Zolpidem	98.7	96.6	93.2	96.3
